# TurboPutative: A web server for data handling and metabolite classification in untargeted metabolomics

**DOI:** 10.3389/fmolb.2022.952149

**Published:** 2022-09-08

**Authors:** Rafael Barrero-Rodríguez, Jose Manuel Rodriguez, Rocío Tarifa, Jesús Vázquez, Annalaura Mastrangelo, Alessia Ferrarini

**Affiliations:** ^1^ Cardiovascular Proteomics Laboratory, Centro Nacional de Investigaciones Cardiovasculares (CNIC), Madrid, Spain; ^2^ Immunobiology Laboratory, Centro Nacional de Investigaciones Cardiovasculares (CNIC), Madrid, Spain

**Keywords:** putative annotations, LC-MS, simplification, lipids, metabolite ID prioritize

## Abstract

Untargeted metabolomics aims at measuring the entire set of metabolites in a wide range of biological samples. However, due to the high chemical diversity of metabolites that range from small to large and more complex molecules (i.e., amino acids/carbohydrates vs. phospholipids/gangliosides), the identification and characterization of the metabolome remain a major bottleneck. The first step of this process consists of searching the experimental monoisotopic mass against databases, thus resulting in a highly redundant/complex list of candidates. Despite the progress in this area, researchers are still forced to manually explore the resulting table in order to prioritize the most likely identifications for further biological interpretation or confirmation with standards. Here, we present TurboPutative (https://proteomics.cnic.es/TurboPutative/), a flexible and user-friendly web-based platform composed of four modules (Tagger, REname, RowMerger, and TPMetrics) that streamlines data handling, classification, and interpretability of untargeted LC-MS-based metabolomics data. Tagger classifies the different compounds and provides preliminary insights into the biological system studied. REname improves putative annotation handling and visualization, allowing the recognition of isomers and equivalent compounds and redundant data removal. RowMerger reduces the dataset size, facilitating the manual comparison among annotations. Finally, TPMetrics combines different datasets with feature intensity and relevant information for the researcher and calculates a score based on adduct probability and feature correlations, facilitating further identification, assessment, and interpretation of the results. The TurboPutative web application allows researchers in the metabolomics field that are dealing with massive datasets containing multiple putative annotations to reduce the number of these entries by 80%–90%, thus facilitating the extrapolation of biological knowledge and improving metabolite prioritization for subsequent pathway analysis. TurboPutative comprises a rapid, automated, and customizable workflow that can also be included in programmed bioinformatics pipelines through its RESTful API services. Users can explore the performance of each module through demo datasets supplied on the website. The platform will help the metabolomics community to speed up the arduous task of manual data curation that is required in the first steps of metabolite identification, improving the generation of biological knowledge.

## 1 Introduction

Untargeted metabolomics aims at measuring the entire set of metabolites in a wide range of biological specimens, in a certain time and under particular conditions (Nicholson et al., 1999; Fiehn, 2001). However, unlike genes, transcripts, or proteins, metabolites are not encoded in the genome but are the result of chemical transformations by enzymes acting on diverse substrates (i.e., diet, drugs, pollutants, other metabolites, and the environment, among others), resulting in a group of molecules with high chemical diversity. Hence, the identification and characterization of each one of the elements of the metabolome remain the major bottleneck of this discipline (Bowen and Northen, 2010; Creek et al., 2014). Moreover, since different sample preparation and analytical methods are used to increase metabolome coverage (Theodoridis et al., 2018), researchers generally deal with massive and complex datasets where not all of the entities are biologically relevant or, even more challenging, identifiable (Baker, 2011). Indeed, it has been reported that only 25% of the observed compounds can be putatively annotated/identified (Baker, 2011). Furthermore, peak tables, which are generated from liquid chromatography coupled with mass spectrometry (LC-MS) analyses [i.e., the most commonly used platform for untargeted metabolomics experiments (Hu et al., 2020)], are composed of redundant entities due to commonly occurring adducts, neutral losses, isotopes, and in-source fragments that eventually correspond to the same metabolite and, thus, largely depend on the analytical method employed rather than on the system studied (Alseekh et al., 2021). This results in an arduous and highly time-consuming data processing step in untargeted metabolomics-based experiments that can lead to wasteful use of resources as well as unintended biases when publishing results.

Over the past years, several computational tools have been proposed to process these complex datasets (O’Shea and Misra, 2020; Misra, 2021). Among them, XCMS, MZmine, MSDial, and OpenMS focus on the data preprocessing step by grouping features arising from the same chemical entity (i.e., adducts, isotopes, and in-source fragments) and allowing peak-picking, data alignment, and noise and background signal elimination (Smith et al., 2006; Forsberg et al., 2018). HERMES uses raw MS1 data to optimize MS2 acquisition, improving the annotation by a molecular-formula-oriented and peak-detection-free method (Giné et al., 2021). Finally, others focus on the identification of metabolites by predicting, through pathway activation, the most probable metabolite identification starting from a list of MS peaks (Pang et al., 2021). However, even though these tools allow reducing data matrix complexity and facilitate compound identification, this latter task remains highly dependent on manual data curation by the researcher, especially in the case of low-intensity peaks and with low MS2 resolution.

The first step for metabolite identification can be achieved by tools such as Ceu Mass Mediator and MetaboSearch that allow searching the experimental monoisotopic mass against databases within a defined mass error window and considering expected adducts/neutral losses (Smith et al., 2005; Gil de la Fuente et al., 2018). However, their typical output is a comprehensive list of potential identifications that has to be manually inspected by the researcher in order to remove redundant information. Indeed, according to the settings included in the search (i.e., error window and adducts) as well as the number of libraries/databases used (more than one is generally preferred), the list of potential metabolites can be extremely large. Specifically, for the same monoisotopic mass entry, the output data table can include several synonyms depending on the nomenclature used in the selected database (i.e., IUPAC vs. common name) as well as highly informative and specific data that cannot be deduced from the monoisotopic mass [i.e., the stereoisomeric distribution of metabolites (Schrimpe-Rutledge et al., 2016) and the double bond position (Groessl et al., 2015), among others]. This exponentially increases the number of possible identifications that are instead attributable to a few less specific metabolic IDs, hindering an already complex dataset. Moreover, depending on the database employed and the biological setting investigated, relevant biological information might be hidden by less likely putative annotations, entailing additional effort from the researcher who has to manually classify each entry.

The list of potential metabolites can be further reduced and the confidence level of the annotation increased by including retention time and/or spectral similarity with commercial/public spectral libraries/databases (Kind et al., 2018). However, only the use of a standard (pure compound), which is analyzed under identical analytical conditions, allows unambiguous identification of annotated molecules, as outlined by the Metabolomics Standards Initiative (Sumner et al., 2007; Schrimpe-Rutledge et al., 2016). This notwithstanding, only a few metabolomics studies comply with this identification confidence level (level 1). On the contrary, majority of the metabolomics studies only report the most probable ID (i.e., metabolites that are putatively annotated according to mass accuracy and spectral similarity, level 3 and level 2) (Alseekh et al., 2021). This results in a demanding and highly time-consuming data curation task for the researcher who has to manually reduce the data table in order to provide a list of the most likely identifications that in turn allows biological interpretation of the data.

Here, we propose TurboPutative (https://proteomics.cnic.es/TurboPutative/), a flexible and user-friendly web-based platform composed of four modules (Tagger, REname, RowMerger, and TPMetrics) that streamline data handling, classification, and interpretability of untargeted LC-MS-based metabolomics data. TurboPutative allows researchers dealing with massive datasets containing multiple putative annotations to reduce the number of entries while classifying them through an automated and customizable workflow. To the best of our knowledge, no other existing web server offers the possibility to considerably simplify the data table with putative annotations without overlooking relevant biological data. TurboPutative facilitates extrapolation of biological knowledge and improves metabolite prioritization for subsequent ID confirmation or pathway analysis. Key features of TurboPutative as well as details about the server and its interface are provided below.

## 2 Methods

**FIGURE 1:**
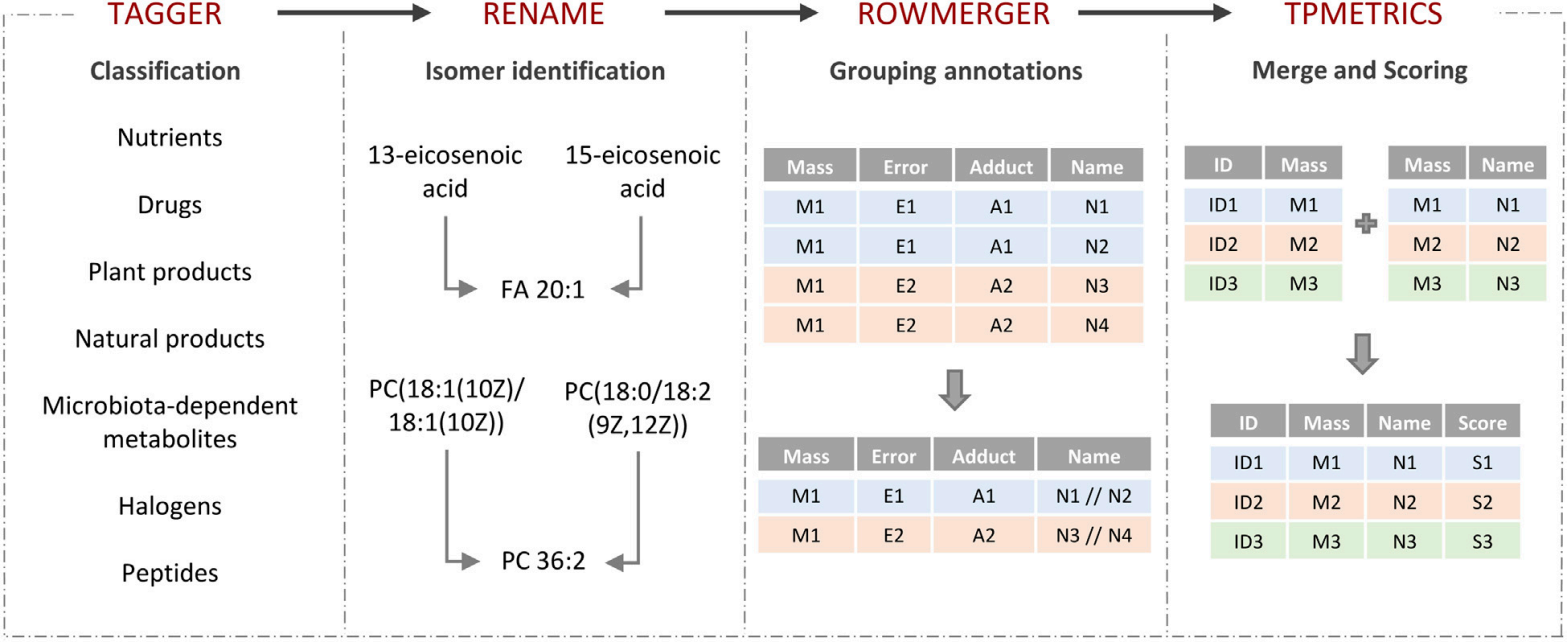
TurboPutative workflow. The complete pipeline is composed of four modules: (i) Tagger, (ii) REname, (iii) RowMerger, and (iv) TPMetrics that can work sequentially or run alone. The table generated by one module includes the simplifications and modifications introduced by the previous one.

Turboputative is a free web server consisting of four modules that can be executed sequentially: 1) Tagger, 2) REname, 3) RowMerger, and 4) TPMetrics (Figure 1). Demo datasets are available for the user to explore the platform, and a working example is provided to show the results of the data curation by carrying out the whole pipeline. In addition to uploading the input file, users can customize the parameters of each module and select which one to execute.

### 2.1 Modules

#### 2.1.1 Tagger

**FIGURE 2:**
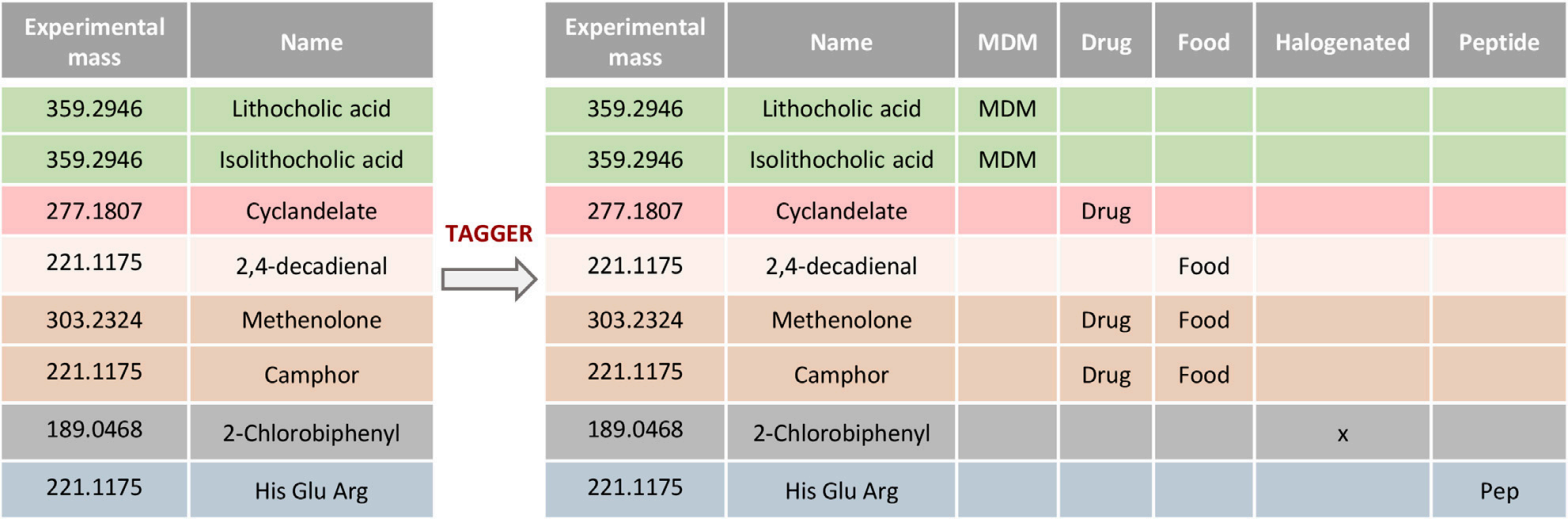
Example of compounds classification using Tagger. Entities retrieved from databases can be classified as nutrients, drugs, microbiota-dependent metabolites (MDM), natural products/plants, halogens, or peptides.

Metabolites are defined as small molecules present in a biological compartment. This definition, however, simplifies the depiction of a more complex environment in which a combination of endogenous compounds and xenobiotics is actually present (Nicholson and Wilson, 2003). Moreover, since the typical output table, which is generated after the first step of the metabolite’s identification, is composed of all the possible annotations that comply with the settings included in the search against databases, the resulting list might include, for the same entity, metabolites of different origin, including xenobiotics (i.e., drugs and their catabolites, contaminants and toxins), metabolites derived from the co-metabolism between the host and the gut microbiota, or endogenous metabolites, among others. In order to reduce this complexity and enhance subsequent metabolite prioritization, we developed “Tagger,” a classification module in TurboPutative. Tagger parses several databases (see below) and classifies the compounds in the dataset according to their origin as nutrients, drugs, microbiota-dependent metabolites (MDM), natural products/plants, contaminants (i.e., halogen-containing compounds), or peptides (Figure 2). This enables narrowing down putative identifications only based on accurate mass search by allowing the user to focus on those molecules either expected to be present in the system or biologically relevant for the study. For instance, if among the different options corresponding to the same chemical entity there are drugs that were not supplied to the system studied, the classification by Tagger allows the user to detect and remove these candidates from the list, making the dataset more manageable. On the other hand, if the researcher is investigating the effect of the gut microbiota on the host metabolism [a hot topic in research (Nicholson et al., 2012)], the MDM classification would pinpoint those metabolites by converting analytical data into biological knowledge as well as prioritizing their subsequent ID confirmation.

The classification is performed using in-house predefined lists of metabolites that were generated by parsing different databases as described in the following:

1) Nutrients (abbreviated as food): the reference list contains compounds that are extracted from the Human Metabolome Database (HMDB 4.0) (Wishart et al., 2018b) being classified as “Food” or as “Food and Nutrition.”
2) Drugs: the reference list is composed of compounds from the DrugBank (Wishart et al., 2018a) and HMDB metabolites that are classified as “Drug” or as “Pharmaceutical industry.”
3) Plants: the reference list contains compounds extrapolated from the PlantCyc (Hawkins et al., 2021) database.
4) Natural products (NPs): the reference list contains compounds extracted from the LOTUS database (https://lotus.naturalproducts.net/).
5) Microbiota-dependent metabolites (MDMs): The reference list contains an in-house curated list of compounds and other metabolites included in the Metabolomics Data Explorer tool that is developed by the Sonnenburg Laboratory (Han et al., 2021).
6) Halogens (x): halogenated compounds are classified by applying regular expressions to the molecular formula (if available from the input dataset) or to the metabolite’s annotation. For example, the compound 6-(2-Chloroallylthio)purine can be classified as halogen by applying the regular expression “(F|Cl|Br|I) (?! [a-z])” to its molecular formula (C8H7**Cl**N4S). Alternatively, the regular expression " ([Ff]luor (?!ene)|[Cc]hlor (?!ophyl)|[Bb]rom|[Ii]od)" can be applied to its name (6-(2-Chloroallylthio)purine).
7) Peptides (Pep): sequence of amino acids reported following the three-letter symbol abbreviation are classified as peptides using a regular expression (e.g., Arg-Lys-Ile).

Of note, only those metabolites that have an exogenous origin are included in the reference list accessed by Tagger. On the contrary, metabolites that are classified as both “Endogenous” and “Food”/“Food and Nutrition”/“Drug”/“Pharmaceutical industry” in the HMDB are not included in the in-house lists. This is the case of metabolites as amino acids that can be present in nutrients/plants/drugs/natural products but also be the result of endogenous metabolism and, thus, are not classified by Tagger. Finally, different nomenclatures are retrieved from the PubChem database for each compound and included in the in-house list, making Tagger classification more inclusive.

#### 2.1.2 REname

The typical dataset generated after the first step of metabolite identification includes multiple options for the same entry. According to the database used, the same metabolite can be reported following different nomenclatures, producing a large list of repetitive annotations of limited utility for the researcher who needs expertise and extensive knowledge to efficiently remove/unify them. Furthermore, the same experimental monoisotopic mass can not only belong to compounds that are biologically different from each other but also to different stereoisomeric forms of the same chemical entity (i.e., same chemical formula but different spatial arrangement). However, unless experiments employing chiral separation, tandem-MS^n^, or standards are employed, isomers cannot be unambiguously identified by mass accuracy alone, and any detail concerning stereoisomerisms/tautomerism would only hinder an already complex dataset. This is particularly relevant for lipids that, independently of their complexity, exist in nature as different isomers (and with diverse combination of isomers) (Cao et al., 2020) but also of those metabolites that have chemical substituents/double bonds.

**FIGURE 3:**
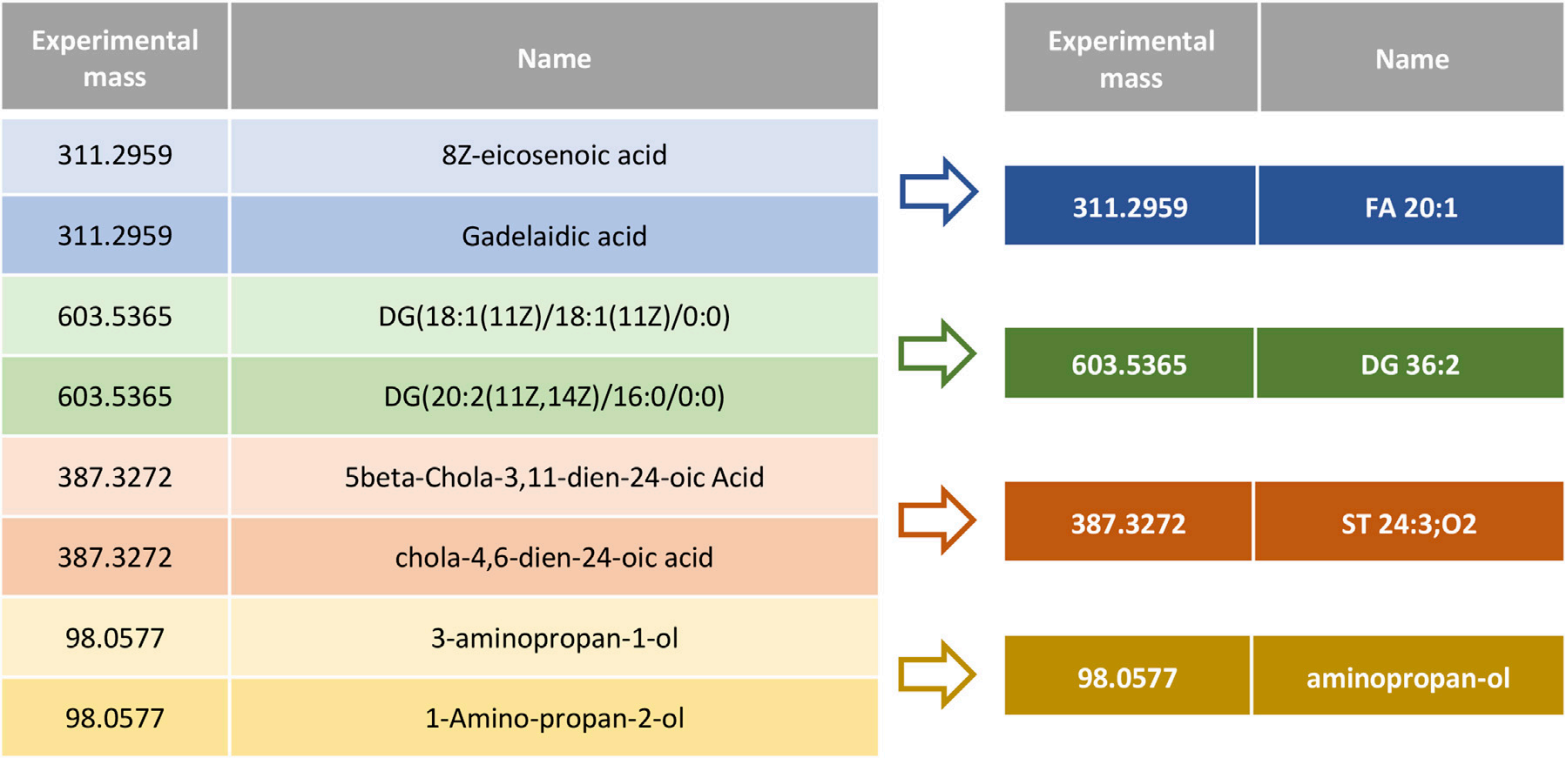
Metabolite name processing using REname. Isomers and metabolites listed with different nomenclature are combined under a common, simplified, and established name or abbreviation.

**FIGURE 4:**
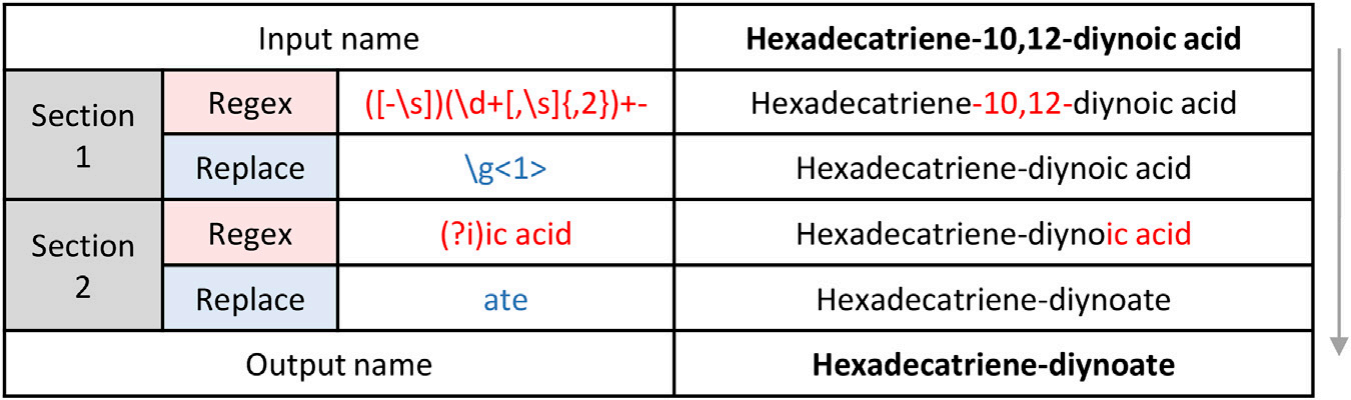
Mechanics of metabolite name processing with regular expressions.

In order to improve data handling and relevant data visualization, we have developed REname, which is able to simplify/abbreviate the names of the compounds, ultimately allowing isomers and equivalent compounds to gain recognition. In the case of lipids, REname uses a dictionary with abbreviations extracted from the Lipid Maps Structure Database (LMSD) (Sud et al., 2007) and the Goslin package (Kopczynski et al., 2020, 2022) to process the compound name, which was obtained in the search against different databases (e.g., LipidMaps, SwissLipids, HMDB) by converting its redundant information into a simpler and abbreviated form. Specifically, REname will extract and use the abbreviation: 1) the head group of lipids (e.g., PE or phosphoethanolamine), and for the fatty acyl chains, 2) total number of carbon atoms, 3) total number of double bonds, 4) type of linkage (i.e., ether or vinyl ether bonds), and 5) presence/amount of hydroxyl and methyl groups (Figure 3). On the other hand, for other metabolites or if no abbreviation is available in the dictionary, REname will process the metabolite name by applying a set of regular expressions that will sequentially process the entry, for example by deleting the position of functional groups or the spatial configuration (Figure 4). Finally, REname will group all entities, that after the processing, have the same abbreviated form and the same accurate experimental mass, removing redundant data. Of note, despite the redundant data removal, the simplification performed by REname does not result in an overall loss of information since unprocessed data tables can always be retrieved at every step of the pipeline.

#### 2.1.3 RowMerger

**FIGURE 5:**
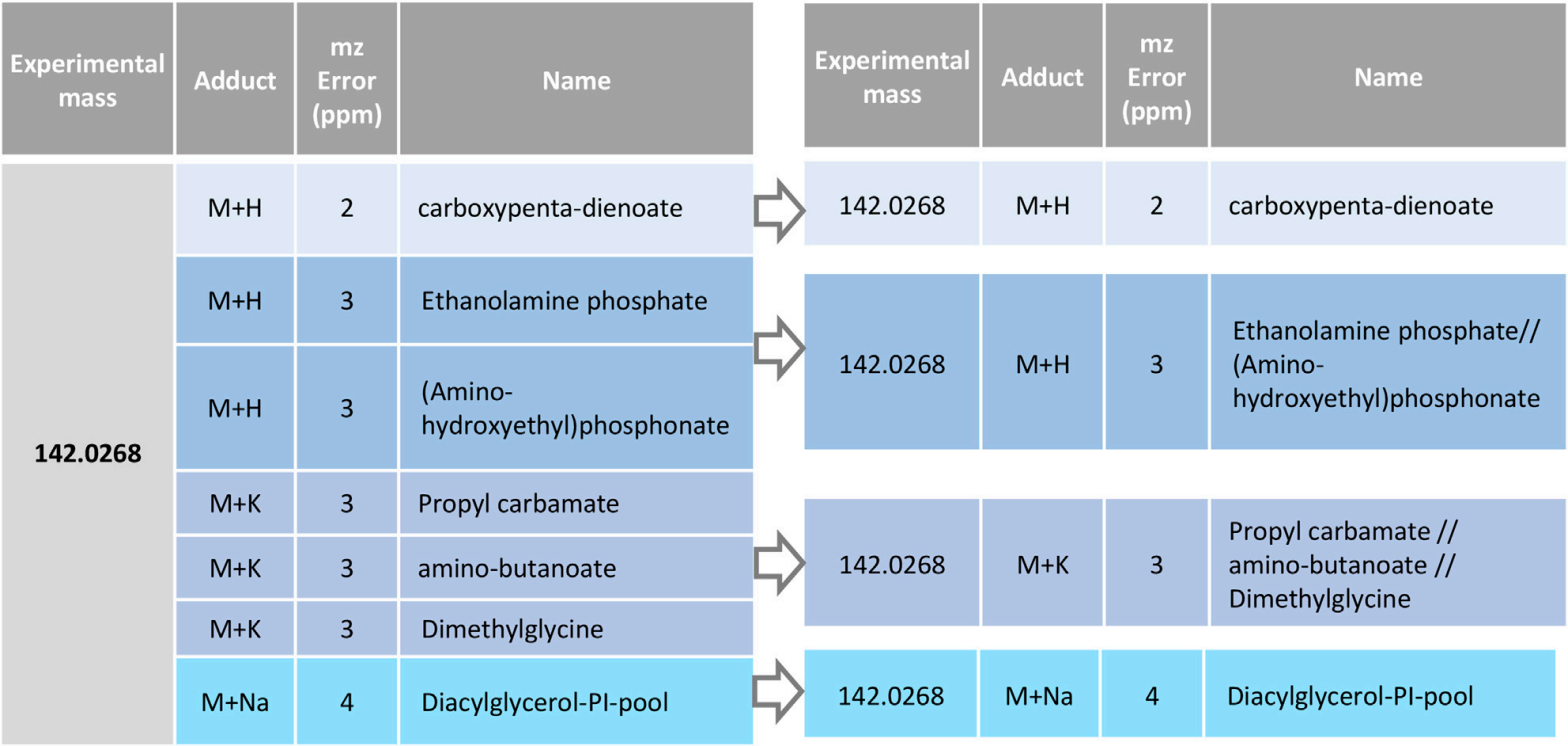
Annotations grouping by RowMerger. Candidate metabolites associated to the same experimental mass and the same adduct and mass error window (ppm) are unified under a single entry.

This module allows grouping the possible annotations by following criteria that are customizable by the user. The user can select which information from the data table will be compared to perform the merging (i.e., the same mass error and/or adduct type, the same Tagger classification, among others) and which one will not be compared (i.e., the abbreviated name). By default, RowMerger will combine entries that have the same experimental mass (both neutral and mass to charge values), adduct, and mass error without processing the abbreviated name, which will be instead grouped with other potential annotations that comply with the criteria selected above and separated by “//” (Figure 5). This will allow a reduction of the dataset, greatly facilitating the comparison among annotations and the visualization/interpretation of the results, as depicted in Figure 5.

#### 2.1.4 TPMetrics

Due to the nature of data generated from LC-MS, metabolites can be detected in the mass spectrometer only as ions (including different charge states, e.g., M+2H) that are generated in the ion source after protonation or deprotonation, neutral losses (M + H − H_2_O or M + H − NH_3_), or in association with metals (e.g., Na+, K+, HCOO−, CH3COO−). Moreover, depending on the nature of the metabolites, more than one adduct is generally formed in the ion source, with only one being more probable and abundant than the others. Thus, several signals coming from the same compound can be present in the final table with putative identification, increasing the complexity of the data interpretation. However, from a biological point of view, only the metabolites present in the biological sample, and not the multiple ion species generated by the MS, are responsible for certain activities or metabolism and thus of interest to the researcher.

Even though several open-source (e.g., XCMS and CAMERA, OpenMS, MZmine, and MSDial) (Li, 2020) or commercially available software (e.g., Agilent MassProfiler Professional, and Thermo Compound Discoverer) are able to cluster together features generated from the same metabolite, not all of them are correctly assigned (Gil de la Fuente et al., 2018). Therefore, all possible adducts are usually considered for the accurate mass search against databases, exponentially increasing the number of resulting annotations. At this point, the researcher is usually bound to manually compare the different options that are assigned to the same entry, in order to prioritize the subsequent steps of ID confirmation or biological interpretation.

In order to facilitate these tasks and enhance the accuracy of the metabolite identification, we have developed TPMetrics that further simplifies the matrix by suggesting the most probable annotation among the multiple options. TPMetrics applies a multi-criteria scoring algorithm that is based on the concept that signals coming from the same molecular entity and those sharing the same biochemical class show a common behavior in the MS and a similar correlation pattern across the samples. Notably, intensity correlations have already been successfully employed to improve annotation and reduce the data set complexity prior to putative identifications as well as to point out lipids that are biochemically connected (Kuhl et al., 2012; Ovčačíková et al., 2016; Köhler et al., 2021).

**FIGURE 6:**
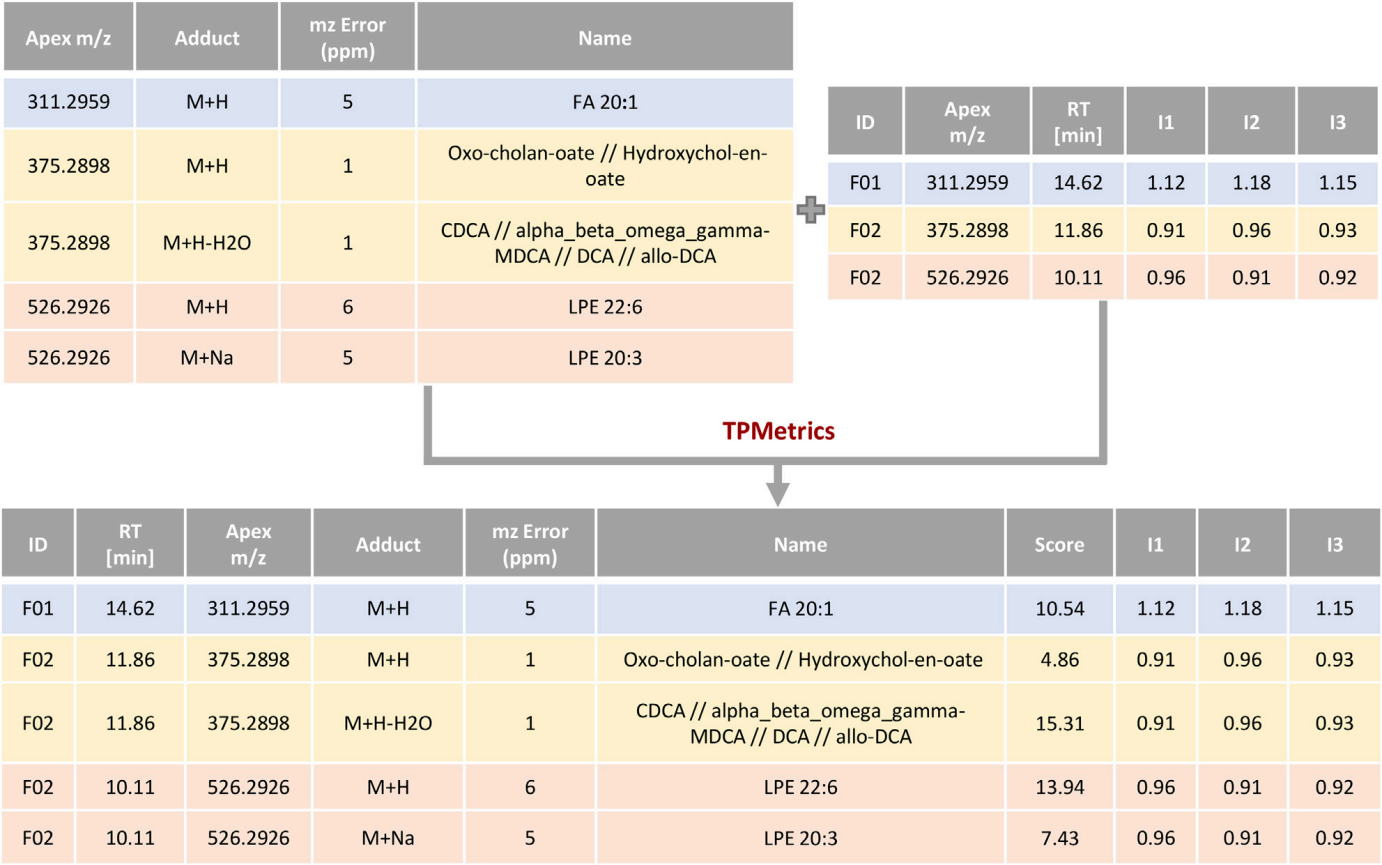
Example of scoring by TPMetrics. Feature annotations are combined with feature intensities and information/metadata useful for the researcher and then scored according to ionization probability and correlation profiles.

TPMetrics first combines the output of RowMerger with the MS1 intensity table that is uploaded within this module. At this stage, the user can also combine additional metadata or other relevant information deemed useful for the interpretation of the results (e.g. *p*-values, fold changes, the metabolite’s mean/median relative abundance) (Figure 6). Subsequently, the algorithm assigns a score based on the probability that a certain metabolite has to form a specific adduct in the MS ionization source. Since this aspect is strictly related to the analytical conditions employed in the study (i.e., the mobile phase composition, the MS polarity mode, among others), the user needs to define and rank the possible adducts from the most to the least probable one within every metabolite class. Next, the algorithm computes a pairwise correlation among those annotations that have the same monoisotopic mass and fall within a narrow user-defined timeframe (e.g., metabolites that are formed with multiple adducts) and generates a score, taking into consideration the highest positive correlation coefficient among the features. Then, it computes an additional score to account for those annotations referring to the same class (e.g., lipid class) by performing a correlation analysis among features’ intensity across samples and within a user-predefined RT window (this time with a higher time frame, set by default at 2 min). TPMetrics also accounts for the mass accuracy by penalizing the annotation mass error and eventually computes the resulting multilevel score. Thus, annotations with higher scores will be preferred over other possible identifications and reported in the final output table of this module.

### 2.2 Input/output

TurboPutative receives an input table containing the putative annotations (e.g., generally the output of the CMM or MetaboSearch) that requires few essential information to be processed, including the mass of the compounds (column name: *Experimental mass*) and the name of the assigned metabolite (*Name*). However, to maximize the use of the different modules, it is also recommended to include (if available) other columns encompassing additional information such as the adducts (*Adduct*) and the mass error (*m/z Error (ppm)*) considered for the metabolite’s annotation as well as the molecular formula (*Formula*) of the putative annotations. TurboPutative accepts tab-separated values (TSV) and excel (XLS, XLSX) formats up to 100 MB and 100,000 entries, whereas the format of the output tables (i.e., either TSV or XLSX formats) can be selected by the user. In the case of executing TPMetrics, the user needs to upload a second table that assigns the intensities of each feature to its corresponding mass (*Experimental mass*) and *RT*. This second table can also contain any other information of interest/choice (e.g., *p* value and fold change). Notably, a sample dataset is downloadable as a template for the user, whereas a working example is provided to show the results of the whole pipeline execution.

TuboPutative generates a table for each executed module. Thus, since they work sequentially, the output table of one module will be the input table of the following one, and so on. When running TPMetrics, an additional filtered table is generated with the most probable annotation for each feature based on the calculated multi-criteria score. In the case of running the workflow via the web server, all input and output tables can either be downloaded or displayed in the browser. In addition, a summary section is included in the browser to depict the results of the complete workflow execution as a graphic that displays the number of rows/metabolites obtained in each output table after the execution of the corresponding module and a histogram indicating the number of metabolites labeled by Tagger.

The platform has been tested with more than 100 input tables, most of them generated by CMM, and ranging from 500 to 30,000 rows. In these ranges, the running time follows a nearly linear increase with the number of rows contained in the input. Of note, there may be fluctuations depending on whether the compounds contained in the table were not processed by the server at any time (they are not cached) or if the table contains compounds that require more complex processing. Considering these variations, a table with 500 entries is processed in approximately 10 s, while a table with 30,000 entries is processed in 250 s. In addition, when comparing the output of TPMetrics with the identification achieved by tandem-MS (MSI level 2), the module is able to assign the highest score to the most probable features with a likelihood of 80%.

### 2.3 Web server

TurboPutative is a web server (https://proteomics.cnic.es/TurboPutative/) based on the web application framework Express (https://expressjs.com/). TurboPutative Web Server was tested using several web browsers: Chrome 100.0.4896.88 (64-bit), Firefox 95.0.2 (64-bit), Safari 13.1.2 (64-bit), and Opera 85.0.4341.47 (x86_64).

The modules were developed using the C++ programming language and integrated into a Python program. When users submit a job, the Python program will be executed in the back-end and a waiting page will be sent to the web client. This page will be refreshed every 10 seconds and, when finished, the server will send the matrix data to the client, displayed in different tables in the browser, and a link to download the results.

TurboPutative is licensed under a Creative Commons Attribution-NonCommercial-NoDerivs 4.0 Unported Licens, and the source code is available on GitHub repository (https://github.com/CNIC-Proteomics/TurboPutative-web).

### 2.4 Web services and command lines

TurboPutative provides web services (https://proteomics.cnic.es/TurboPutative/webservices) for automatic access to workflow execution. The TurboPutative Web Services make use of standard HTTP method calls, often termed RESTful services, and then the HTTP request methods GET and POST can be used to send and receive queries and data. These web services have been developed as asynchronous services; the user can continue their work without interruption, and will be notified when the asynchronous response is returned. Specifically, when a job is submitted, the user gets a job identifier that allows to check the job status and, if applicable, download the results.

RESTful services can be accessed using universal Resource Locators (URLs) or from a command-line (using CURL). A client script in Python language has been provided to show how to use TurboPutative Web Services with a language capable of making standard HTTP requests. In addition, we developed a friendly interface using Swagger (https://swagger.io/) that allows interaction with the TurboPutative Web Services.

## 3 Results and discussion

To illustrate how Turboputative enhances and facilitates the visualization of relevant data generated from an untargeted LC-MS-based metabolomics experiment, we have selected the study of Lorenzo et al. (2021) that is aimed to decipher the role of the humoral immune response mediated by B cells in the development of atherosclerosis.

Atherosclerosis is a silent chronic inflammatory disease that develops over the years and eventually results in sudden and often fatal cardiovascular events, including ischemic heart disease or stroke (Hansson, 2005). Due to its long asymptomatic phase, subjects at higher risk of developing the disease are often underrated, making early diagnosis and the understanding of the underlying mechanism a compulsory need. The presence of antibodies in atherosclerotic plaques has suggested that the immune response is involved in the pathogenesis of the disease (Reardon et al., 2001; Song et al., 2001; Hansson and Hermansson, 2011). However, little is known about the response and influence of the underlying antigenic triggers during atherosclerosis. In this study, Lorenzo et al. (2021) found that an atherosclerosis-generated antibody, A12, induces strong plaque reactivity in an atherosclerosis mice model (Ldlr^−/−^ HFD), and that the distribution and expression of its antigen ALDH4A1 is altered during atherosclerosis. Untargeted lipidomics was then performed to study the effect on lipid metabolism of serial intravenous injections of A12 antibody on the liver. After statistical data analysis, 215 monoisotopic masses were found to be significantly altered and subsequently annotated using Ceu Mass Mediator 3.0 (CMM) (Gil de la Fuente et al., 2018). The CMM output table contained 14,829 potential metabolite annotations.

**FIGURE 7:**
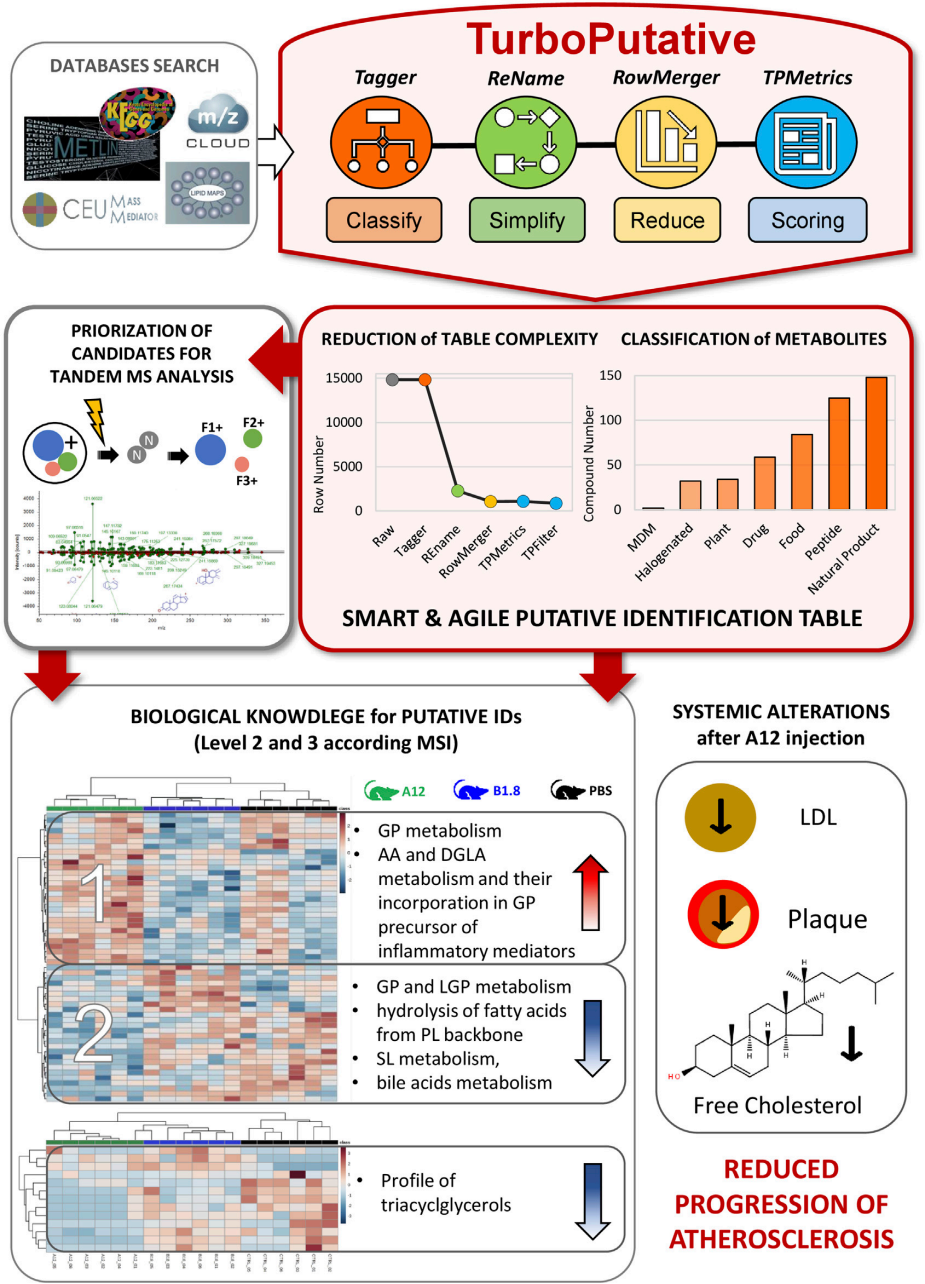
An application of TurboPutative to identify changes in lipid metabolism by A12 antibodies in the progression of atherosclerosis. After databases search, raw putative annotations have been processed with TurboPutative and a simplified and easy-to-use list of entities has been generated. Tagger could classify a total of 420 potential metabolites, whereas the complete workflow has reduced the dataset size by 92%. This has enabled prioritizing the most probable candidates for tandem MS analysis and to explore the biological changes occurring in mice liver after antibodies administration. Hierarchical clustering analyses performed with two list of identified metabolites (confidence identification level 2 and 3 according to MSI) has highlighted several alterations in lipid metabolism. These findings corroborate the hypothesis that A12 antibody delays plaque formation and may reduce the progression of atherosclerosis and improve circulating lipid profiles.

An alpha version of TurboPutative was used to handle this big dataset, and the four modules (Tagger, REname, RowMerger, and TPMetrics) were run sequentially. This reduced the original dataset up to 92% thereby facilitating the subsequent analyses and the generation of biological knowledge from the analytical data (Figure 7). Specifically, Tagger could classify a total of 420 potential metabolites as peptides (125), halogen-containing molecules (32), microbiota-dependent metabolites (2), drugs (59), nutrients (84), plants (34), and natural products (148). This made it possible to discard potential annotations referring to drug/nutrients/halogens that were not supplied to the system as well as identifications that did include peptides or MDM due to their limited relevance for this study. Next, REname conspicuously reduced the size of the table from 14,829 to 2,275 entries (84% reduction), by grouping into unique entries the isomers that could not be distinguished by accurate mass alone, as well as the metabolites reported following different nomenclature in the databases were parsed by CMM. This made the dataset easier to handle while improving the visualization of relevant data, especially in the case of lipids. RowMerger further reduced the dataset size by 92% (i.e., from 2,275 to 1,059 entries) by combining the entries that fell within the same mass tolerance window after database search (i.e., the same mass error and adduct type). Finally, TPMetrics combined the resulting data table with additional information of interest for the researcher (e.g., identifiers and statistical values) and filtered the identification based on the calculated multi-criteria score, thus facilitating subsequent data identification and interpretation.

After TuboPutative data curation, an easy-to-handle list of the most probable annotations was obtained that enabled a quick prioritization of possible lipids to target in a subsequent tandem MS analysis, making this step more efficient. Specifically, 55 lipids were annotated according to their similarity with spectral databases [confidence identification level 2 (Sumner et al., 2007)]. Furthermore, the processing performed by TurboPutative allowed the inclusion of MS1 data that is usually overlooked or incorrectly reported in the final table, providing a more global insight into the biological system studied and allowing the use of majority of the data for the biological interpretation of the results. In this regard, 54 lipids were annotated by accurate mass, retention time, and isotopic pattern distribution (level 3) and considering the consistencies in the direction of change among the different lipids belonging to the same biochemical class (Figure 7).

Notably, although the lipidomics data did not meet the identification confidence level required for unambiguous identification, the results obtained by the lipidomics study clearly showed that A12-treated mice underwent an increase in glycerophospholipids incorporating to the fatty acid chains’ residual arachidonic acid or dihomo-gamma linolenic acid moieties (well-known precursors of inflammation mediators) and a generalized decrease in sphingolipids, bile acids, lysophospholipids, and triglycerides, suggesting a strong effect of A12 on inflammation and lipid metabolism in the liver (Figure 7). These results were consistent with other assays which showed that A12 delays plaque formation, suggesting a therapeutic use of anti-ALDH4A1 A12 antibody to reduce the progression of atherosclerosis and improve circulating lipid profiles.

## 4 Conclusion

Here, we present TurboPutative, a freely available web server tool that, with four simple and customizable steps, streamlines data handling, classification, and interpretability of untargeted LC-MS-based metabolomics data. Despite its ability to considerably simplify the data table with putative annotations, TurboPutative has some limitations that stem from the resources employed in each module. For instance, the classification performed by Tagger might be flawed by the incompleteness of metabolite databases, and some specific annotations (i.e., infrequent common names) that are neither recognized by Goslin or regular expressions, nor included in the LipidMaps database, and may not be abbreviated by REname. To minimize these limitations, the list of classified metabolites and the abbreviation table that is used by TurboPutative will be updated periodically. Moreover, although Turboputative is designed to deal with high-resolution MS data and has been conceived to handle untargeted LC-MS-based metabolomics data, it can also be used to curate data generated from a wide range of mass resolution instrumentation as well as from other soft ionization MS-based platforms (i.e., capillary electrophoresis coupled to MS). This web service tool will be further expanded in the future by integrating new metabolite databases to improve the classification of putative annotations as well as by entailing information on the metabolic pathway associated with the candidate metabolites in order to further facilitate the interpretation of the results.

## Data Availability

Publicly available datasets were analyzed in this study. These data can be found at: https://www.metabolomicsworkbench.org/data/DRCCMetadata.php?Mode=Project&ProjectID=PR000985.
