# Supplementary material for: TurboPutative: A web server for data handling and metabolite classification in untargeted metabolomics
Source: Front Mol Biosci. 2022 Sep 8;9:952149. doi: 10.3389/fmolb.2022.952149 (PMC9493301; doi:10.3389/fmolb.2022.952149)
Supplement: Supplementary file 1 [file DataSheet1.docx]

Supplementary Material

# Web Server Help

TurboPutative can be used from the browser in three simple steps.

## Go to module selection


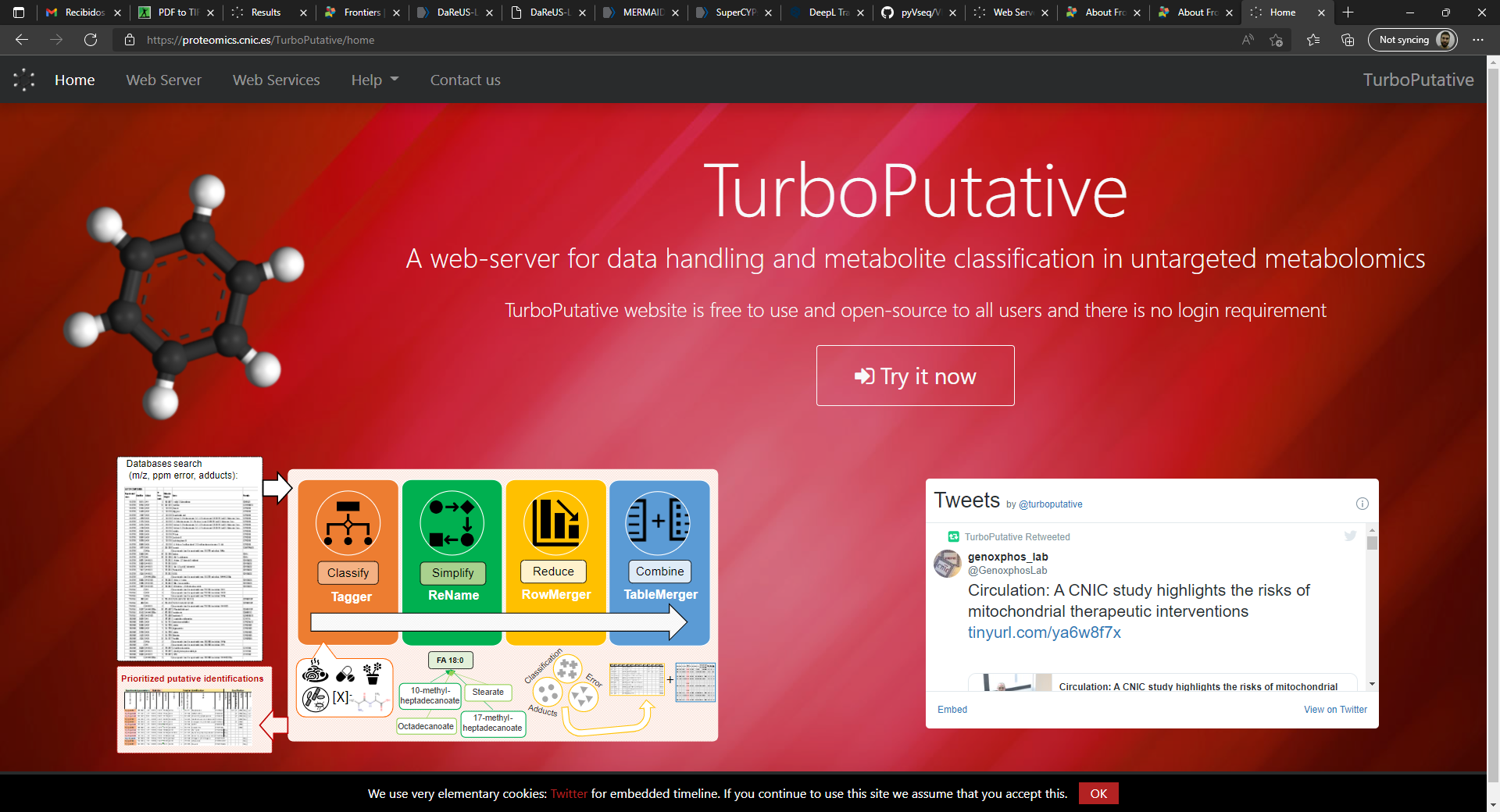


**Supplementary Figure 1.** The Web Server page must be accessed via the navigation bar or via the *Try it now!* button on the homepage.

## Select modules


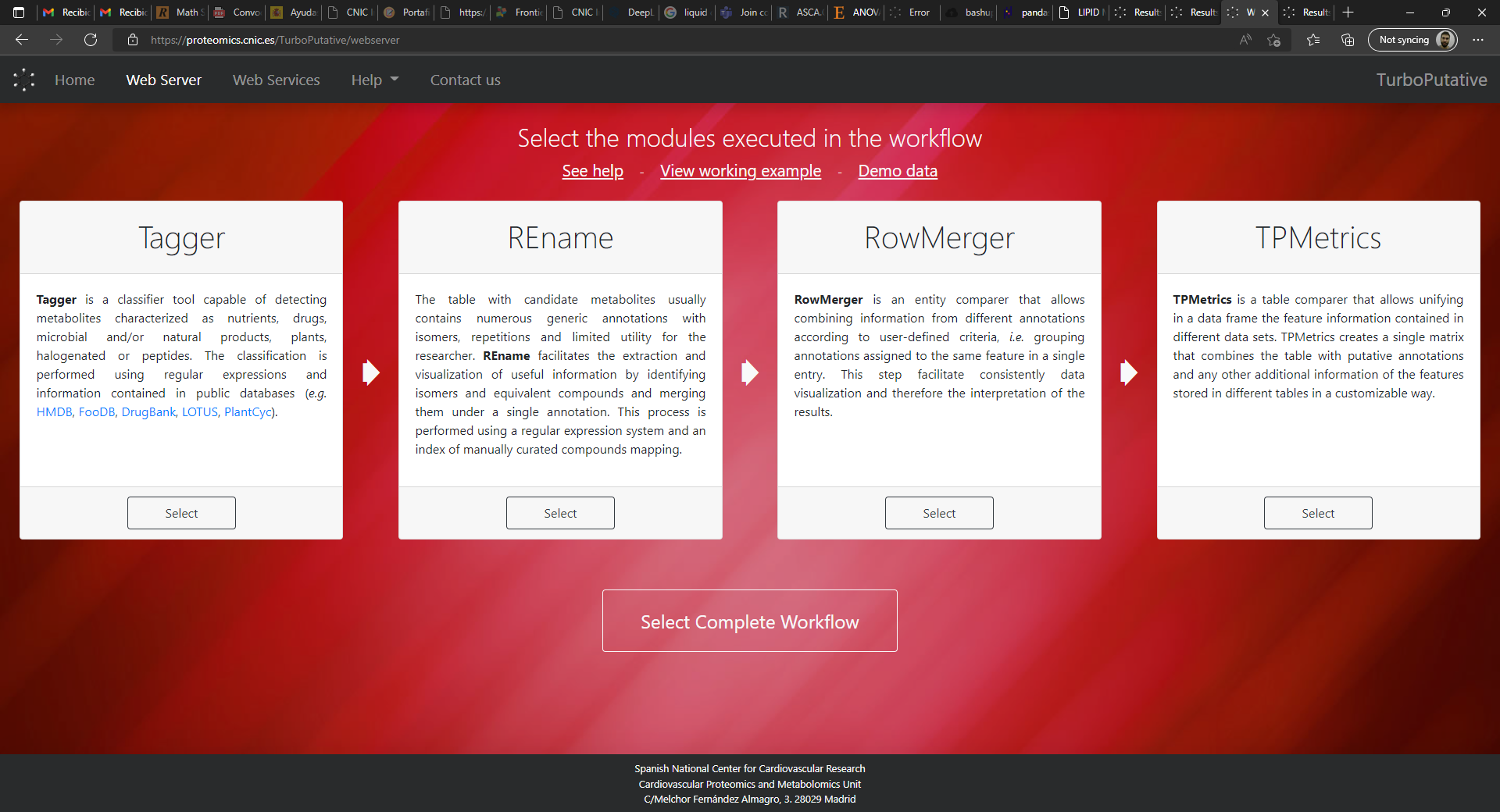


**Supplementary Figure 2.** The executed modules must be selected in the Web Server section. Although not all modules have to be selected, the order of execution is Tagger, REname, RowMerger and TPMetrics.

## Customize parameters


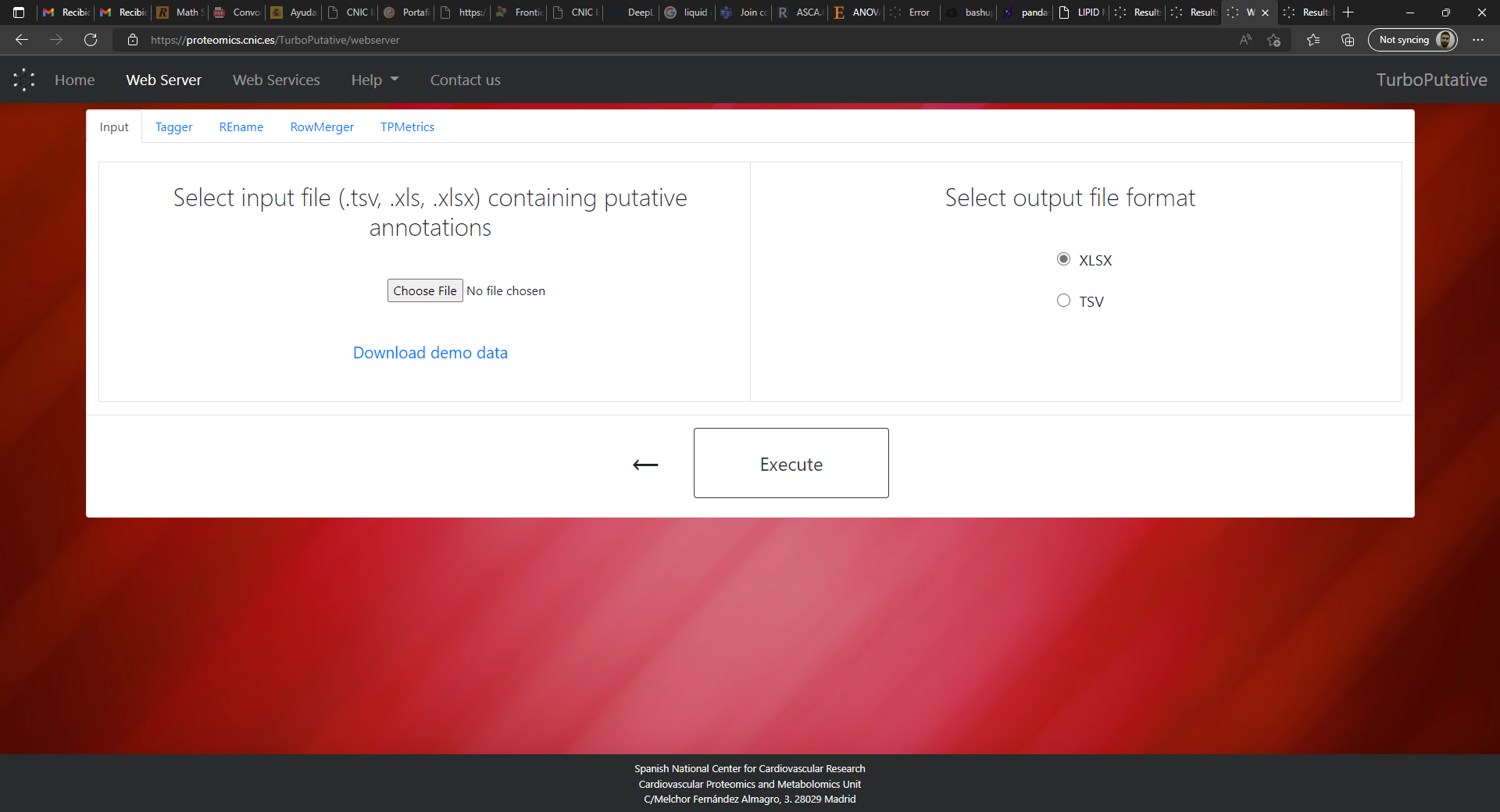


**Supplementary Figure 3.** The file containing putative annotations has to be uploaded and the parameters configured.

## View results


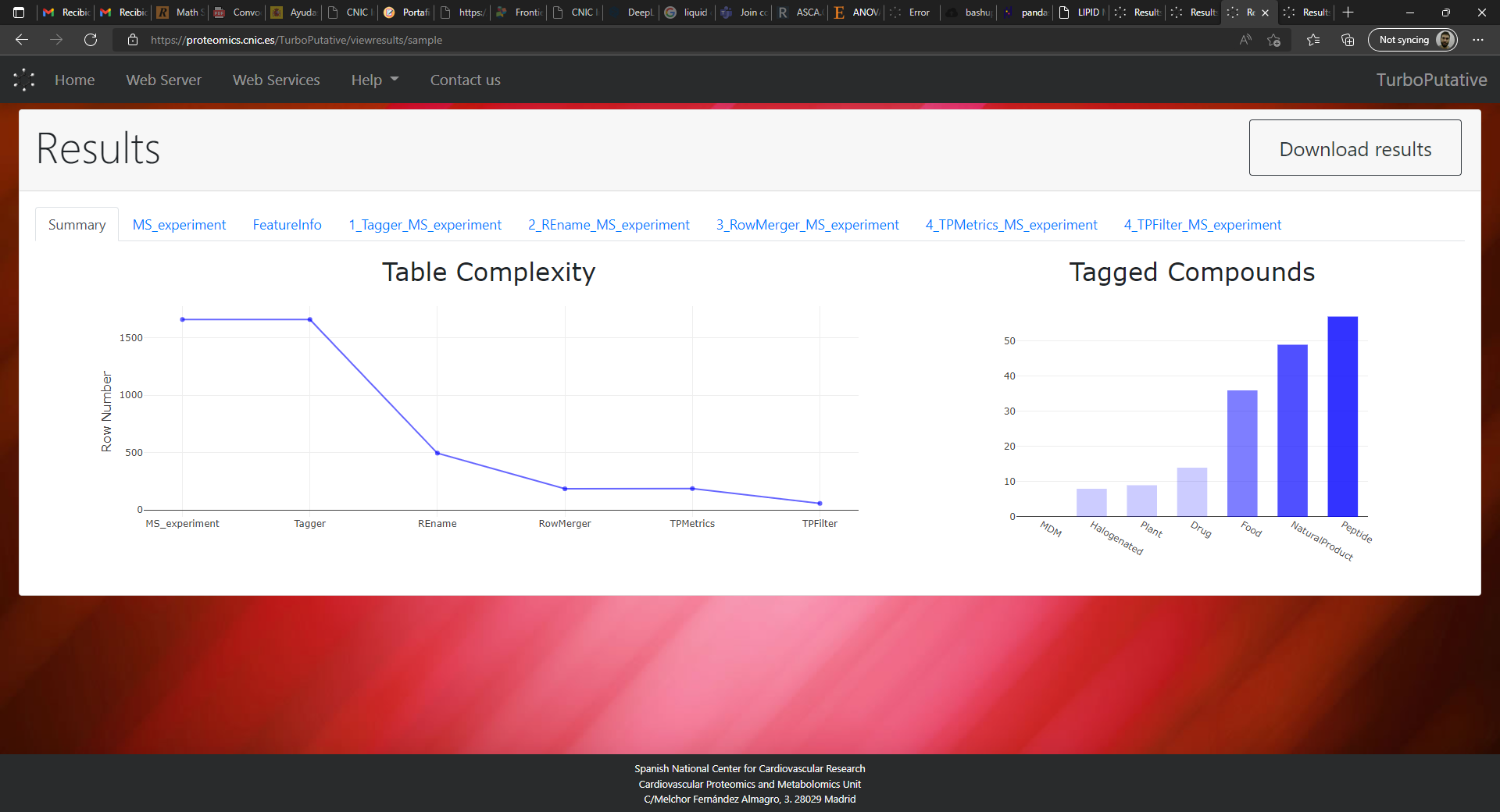


**Supplementary Figure 4.** After the execution a result section is displayed containing summary plots and the processed tables.

# Web Services Help

TurboPutative provides several application programming interfaces (APIs) to send jobs and query data programmatically. These utilities can be used from the browser or by means of a Python script developed for this purpose. The four basic operations that can be carried out are: i) the execution of a customized workflow, ii) the execution of a specific module, iii) the processing of the name of the compounds and iv) the compound classification.

## Execute workflow


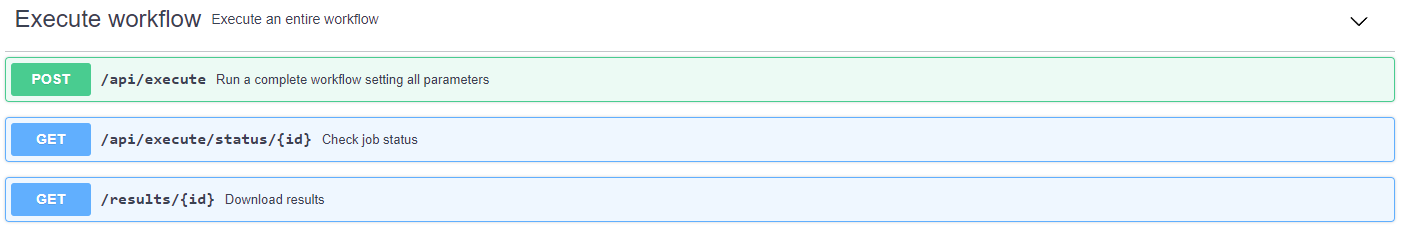


**Supplementary Figure 5.** The "Execute workflow" section allows executing a complete workflow by customizing each of the parameters. To do so, it is necessary to upload the table with the putative annotations and, optionally, a json file with the value of the parameters. The status of the submitted job can be checked using the "status" path, indicating the job identifier. When it is READY, the result can be downloaded using the "result" path and the job identifier.

## Execute module


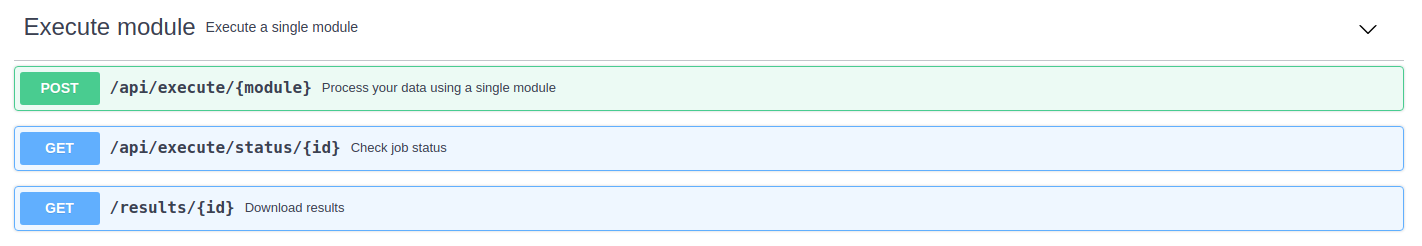


**Supplementary Figure 6.** The “Execute module” section works in a similar way to “Execute Workflow”. However, in this case, only a single module will be executed, which must be indicated in one of the parameters.

## Parse compounds


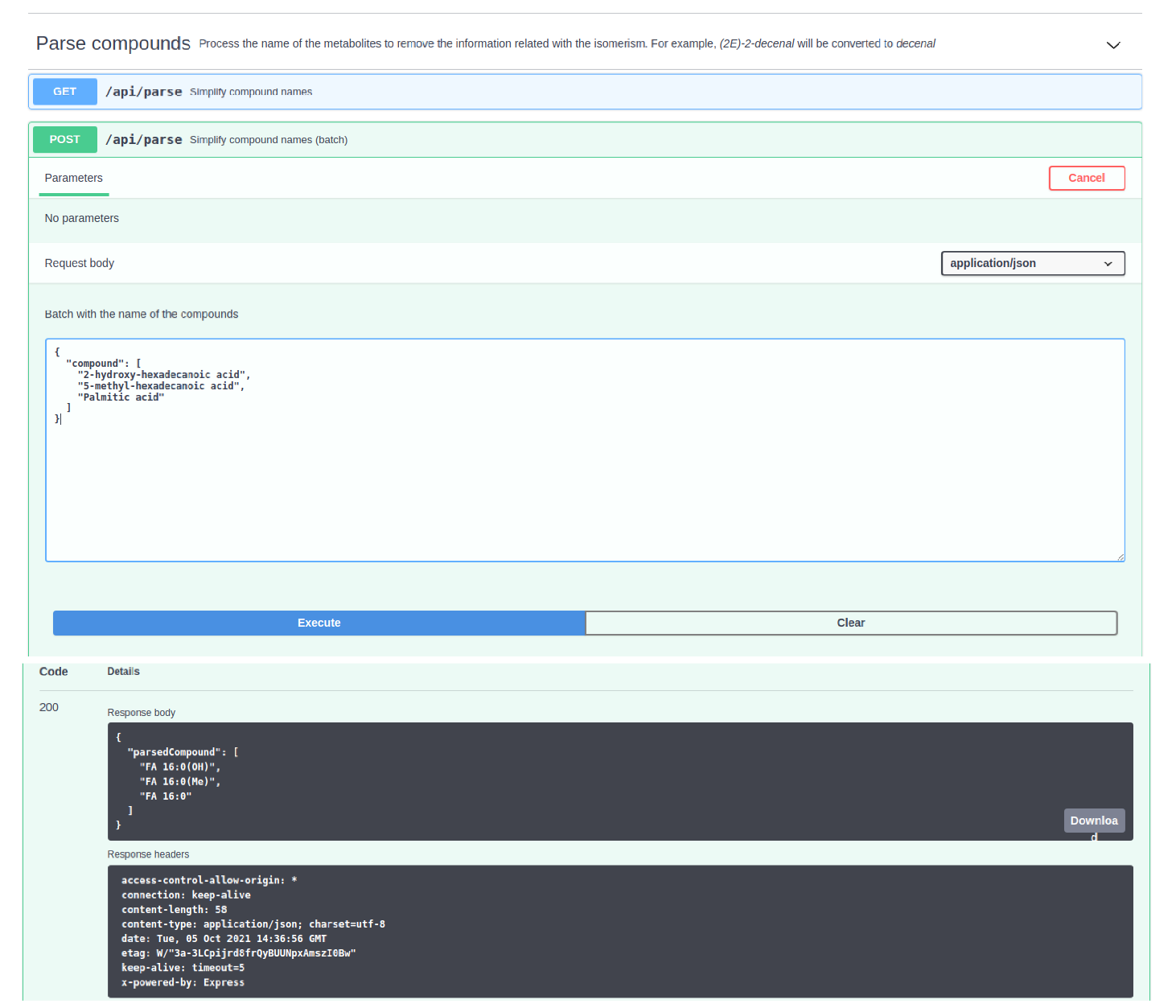


**Supplementary Figure 7.** The "Parse compounds" option simplifies the name of the compounds to facilitate their classification and the identification of isomers.

## Classify compounds


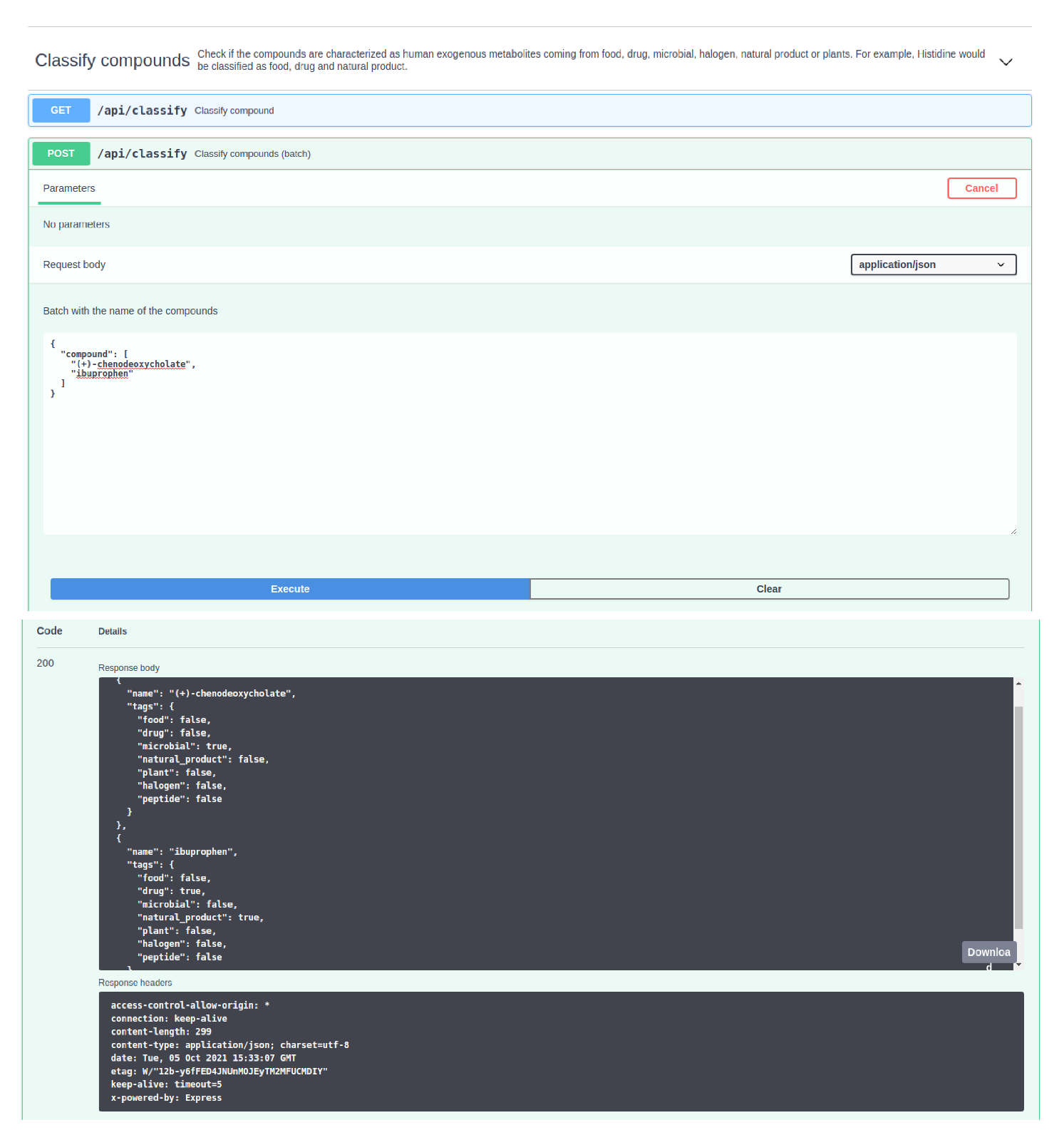


**Supplementary Figure 8.** The "Classify compounds" section is capable of detecting metabolites that can be classified as nutrients, drugs, microbiota-dependent metabolites, natural products, plants, halogens or peptides.

# TPMetrics Algorithm

Let T be a table with features $f_{1}, f_{2}, \ldots, f_{n}$. For each feature we will consider two properties:

- Retention time: $RT(f_{i})$
- Intensities: $I\left( f_{i} \right)=(I_{1},\ldots,I_{s})$

Each feature $f$ has an associated collection of annotations: $Ann(f) =(a_{1},\ldots,a_{m})$. For each annotation $a$ associated to a feature $f$ we will consider the following properties:

- Molecular weight: $MW(a)$
- Lipid class: $K\left( a \right)$
- Adduct: $Adduct\left( a \right)$
- Mass error: $Err\left( a \right)$

The metric calculated for each annotation $a$ associated to a feature $f$ is derived from the probability of adduct formation and the correlation of $f$ with other features.

**Adduct-associated score:** $\boldsymbol{S}^{\boldsymbol{\alpha}}$

Each lipid class $k$ has an ordered set of adducts associated with it (user defined): $\{\beta_{1}, \ldots, \beta_{n}\}$. We denote by $|k|$ the number of adducts associated to $k$; and by $r_{\beta}^{k}$ the ranking of $\beta$ in the ordered set of adducts associated to $k$.

Consider the annotation $a$ with the lipid class $k=K(a)$ and the adduct $\beta=Adduct(a)$. If $\left| k \right|=1$, the adduct-associated score will be $S^{\alpha}=S_{max}$. If $\left| k \right|>1$, the score will be given by the linear decreasing function $S:\left[ 1, \left| k \right| \right]\to[S_{min}, S_{max}]$:

$$S\left( r_{\beta}^{k} \right)=-\frac{S_{max}-S_{min}}{\left| k \right|-1}\cdot(r_{\beta}^{k}-1)+S_{max}$$

Therefore, $S\left( 1 \right)=S_{max}$ and $S\left( \left| k \right| \right)=S_{min}$. We consider $S_{max}=12$, which is the approximate score that would be assigned to an annotation correlated with two features, with $pvalue={10}^{-5}$ and $r=1$ (see below). On the other hand, we consider $S_{min}=6$, which is the approximate score that would be assigned to an annotation correlated with two features, with $pvalue={10}^{-5}$ and $r=0.5$ (see below).

**Correlation-associated score:** $\boldsymbol{S}^{\boldsymbol{w}}$ **and** $\boldsymbol{S}^{\boldsymbol{k}}$

Two sets of features, $FW$ and $FK$, will be assigned to each annotation based on its molecular weight and lipid class, respectively.

Let $a$ be an annotation belonging to feature $f$. To obtain the sets $FW$ and $FK$ we follow the following rules:

- A feature $f_{i}$ belongs to $FW$ if:
  - There exists some $a_{ij}$ belonging to $Ann(f_{i})$ such that $MW\left( a_{ij} \right)=MW(a)$
  - $|RT\left( f \right)-RT\left( f_{i} \right)|\leq t_{w}$
- A feature $f_{i}$ belongs to $FK$ if:
  - There exists some $a_{ij}$ belonging to $Ann(f_{i})$ such that $K\left( a_{ij} \right)=K(a)$
  - $\left| RT\left( f \right)- RT\left( f_{i} \right) \right|\leq t_{K}$

After obtaining the sets $FW$ and $FK$ the correlation between $I(f)$ and $I\left( f_{i} \right)$ is calculated for all $f_{i}$ belonging to $FW$ (keep positive correlations only) and $FK$ (take absolute values):

- $CorrW^{'}= \left\{ Spearman\left( I\left( f \right), I\left( f_{i} \right) \right) | f_{i}\in FW \right\}$

$CorrW=\{ c | c \in CorrW^{'}, c>0 \}$

- $CorrK^{'}= \left\{ Spearman\left( I\left( f \right), I\left( f_{i} \right) \right) |f_{i}\in FK \right\}$

$CorrK = \left\{ \left| c \right| | c \in CorrK' \right\}$

From the correlations we calculate two scores for each set. Let us look at the case of $FW$ ($FK$ is calculated in an analogous way):

- $S_{1}^{w} = -log \left[ pvalue\left( Max\left( CorrW \right) \right) \right]\cdot Max(CorrW)$
- $S_{2}^{w} = -log \left[ pvalue\left( Sum\left( CorrW \right) \right) \right]\cdot Sqrt\left( \left| FW \right| \right)\cdot Mean(CorrW)$

Where $|FW|$ is the number of (positively) correlated features. Because we calculate two scores, an annotation with two correlations (*e.g.* $\{0.9, 0.5\}$) will have higher score than another annotation with correlations $\{0.7, 0.7\}$ and than another annotation with single correlation $\{0.9\}$.

The p-values are calculated empirically. A set of features $f’_{1}, f’_{2}, \ldots f’_{n}$ with intensities taken at random from the initial table is generated. The distribution of correlations under the null hypothesis ($H_{0}$) is obtained from the correlation matrix of the features $f'$. The process is repeated until 100,000 correlations values are obtained:

$$pvalue\left( c \right)=\Pr\left( |C|\geq|c| \right|H_{0})$$

The scores of each set are then unified:

- $S^{w}=S_{1}^{w}+S_{2}^{w}$
- $S^{k}=S_{1}^{k}+S_{2}^{k}$

**Final Score:** $\boldsymbol{S}_{\boldsymbol{TPM}}$

Finally, the scores are unified by introducing the correction for mass error:

$$S_{TPM} = \left( 1 - E\left( \varepsilon\right) \right)\cdot(S^{w}+S^{k}+S^{\alpha})$$

$E$ is the function that assigns a penalty ratio to each mass error $\varepsilon=Err(a)$:

$$E: \left[ 0, \varepsilon_{m} \right]\to[0, 0.25]$$

$$E\left( \varepsilon\right)= 0.25\cdot\left( \frac{\varepsilon}{\varepsilon_{m}} \right)^{2}$$

Where $\varepsilon_{m}$ is the maximum error considering all the annotations in the table.
